# Human contact in internet-based interventions for depression: A pre-registered replication and meta-analysis of randomized trials

**DOI:** 10.1016/j.invent.2023.100617

**Published:** 2023-03-31

**Authors:** Tobias Krieger, Oliver Thomas Bur, Lenny Weber, Markus Wolf, Thomas Berger, Birgit Watzke, Thomas Munder

**Affiliations:** a Institute of Psychology, University of Bern, Switzerland; b Department of Psychology, University of Zurich, Switzerland

**Keywords:** Depression, Randomized trial, Guidance, Diagnostic interview, Degree of contact, Meta-analysis

## Abstract

**Introduction:**

Internet-based self-help interventions have shown to be effective in the treatment of depression. Several meta-analyses indicated that human contact has a crucial impact on adherence and outcome. While most research focused on the role of guidance during interventions, a review by Andersson and Johansson (2012) suggested that contact before the intervention too may play an important role.

**Objective:**

We investigated the impact of the degree of contact in internet-based interventions (IBIs) for depression on outcome in adults suffering from elevated symptoms of depression.

**Methods:**

We conducted a preregistered meta-analysis (www.osf.io/4mzyd) and included trials comparing IBIs for depression against control conditions (treatment as usual [TAU] or waiting list [WL]) in patients with symptoms of unipolar depression searching the databases PsycINFO and Cochrane's Central Register of Controlled Trials (CENTRAL) limited to entries from EMBASE and PubMed. Following Andersson and Johansson (2012), contact before an intervention was defined as having had a diagnostic interview before the IBI, and contact during intervention was defined as having received guidance during the IBI. IBIs were grouped as providing (0) no contact, (1) contact before the IBI, (2) contact during the IBI, or (3) contact both before and during the IBI. The primary outcome was standardized mean difference (SMD) of the IBI and control in depressive symptoms at treatment termination. Secondary outcomes were study dropout and adherence to the IBI.

**Results:**

We included 56 eligible trials that randomized 13,335 patients to 75 internet-based intervention conditions and control groups (TAU in 23 trials, WL in 33 trials). In total, 44 trials (78.57 %) were judged to show some concerns or a high risk of bias. Overall heterogeneity was high regarding the primary outcome (*I*^2^s < 66 %) and even higher for secondary outcomes (*I*^2^s < 91 %). Degree of contact was a robust predictor of outcome and adherence in all pre-registered and exploratory analyses. We found the effect of the IBI to increase with higher degree of contact. However, in pair-wise contrasts, only IBIs offering both contact before and during the intervention (SMD = 0.573, 95 % CI: 0.437, 0.709) significantly outperformed interventions offering no contact (SMD = 0.224, 95 % CI: 0.090, 0.340).

**Conclusions:**

The results suggest that contact before and during an intervention increases the effects of IBIs. The combination of contact before and during the intervention seems to a pivotal role regarding adherence as well as treatment outcome for patients suffering from depression.

## Highlights

• Degree of contact is a robust predictor of outcome and adherence in internet-based interventions (IBIs).
• Contact before the intervention as well as contact during the IBI are independently associated with outcome.
• Only IBIs with contact before and during the intervention significantly outperformed interventions without any contact.
• Synchronous contact before an internet-intervention seems to play a pivotal role.

## 1 Background

### 1.1 Depression and access to empirically validated treatments

Depression is a highly prevalent public health issue and a debilitating condition for the individual, the healthcare system, and society (Lim et al., 2018). Despite efficacious treatments, many individuals remain untreated and do not get access to empirically validated treatments or at least minimally adequate treatment (Moitra et al., 2022). Internet-based interventions (IBIs) for depression bear the potential to bypass several barriers associated with traditional face-to-face treatments. Potential advantages include location- and time-independent accessibility, a high degree of anonymity and privacy, and easier scalability.

### 1.2 Internet-based self-help interventions for depression

IBIs usually consist of multiple sessions or modules which can be accessed via computer or smartphone. Users can work with the content independently and according to individual time preferences. The programs are usually disorder-specific and often based on adaptations of established self-help protocols or face-to-face treatment manuals. Programs are primarily text-based, often supplemented with audio and video elements, and typically contain some exercises for self-practice. The intervention duration tends to be pre-determined and is typically around 6 to 12 weeks. Since their inception at the end of the 1990ies, research has proliferated, and such programs have been investigated in >300 randomized controlled trials (for an overview on IBIs see Andersson and Berger, 2021). A comprehensive meta-analysis of 83 trials (Moshe et al., 2021), yielded a significant medium overall effect size (*g* = 0.52) for IBIs for depression when compared to any control condition.

### 1.3 The role of human contact

One important distinguishing aspect of IBIs is the amount and type of human contact with health professionals (a) before the intervention and/or (b) during the intervention.

#### 1.3.1 Contact before the intervention

Internet interventions differ in whether they include professional contact before the actual intervention begins. Contact before an IBI usually consists of a structured diagnostic interview in which information relevant for clinical decision-making (i.e., is the intervention suitable for a given person) and/or for study inclusion (i.e., does a given person fulfill all inclusion criteria) is gathered reliably and validly. However, beyond their function in gathering information, diagnostic interviews are also believed to have a facilitating effect. For example, in a study on the acceptance of diagnostic interviews in face-to-face treatments, Suppiger et al. (2009) found that the interview made patients feel taken seriously.

Only a few studies have experimentally tested a pre-treatment interview's effects on outcome. In a recent randomized factorial design study (Bur, Krieger, et al., 2022) in people with increased depressive symptoms, a diagnostic interview did not have an effect on outcome (for more details, see also below). In studies with other patient populations, results are mixed. One randomized controlled trial contrasted the effects of an IBI for social anxiety with and without a pre-treatment diagnostic interview (Boettcher et al., 2012). Although the diagnostic interview had no additional effect on anxiety, it influenced secondary outcomes (depressive symptoms and general distress) and patient adherence. In contrast, a recent study using a factorial design investigated whether a diagnostic interview has an impact in an IBI for people with alcohol misuse (Sundström et al., 2022). Results indicated that a pre-treatment assessment interview did not improve drinking outcomes. The authors argue that the lack of effect may be because in the online clinic where the study was conducted, some degree of human contact with all participants, regardless of group assignment, was provided by default.

#### 1.3.2 Contact during the intervention

Contact during the intervention is often referred to as guidance, and interventions are broadly categorized as guided or unguided (also called “self-guided”), depending on whether some form of contact during the intervention is provided. Unguided interventions are completed entirely independently by patients without any contact. These interventions can reach many people simultaneously but are often associated with high dropout rates (Christensen et al., 2009). In guided interventions, patients are accompanied by professionals or trained laypersons who offer minimal support. Via the internet, it is possible to communicate synchronously in real-time (e.g., via telephone, chat, or videoconferencing) or asynchronously (e.g., through email on a closed, secure platform). IBIs often select asynchronous tools, such as secured emails, for guidance as it allows temporal flexibility for patients and therapists. However, there are also remote interventions where guidance takes place in a synchronous manner, e.g., via phone or videoconferencing. Content-wise, the individualized feedback aims at increasing patient adherence (i.e., program use) by having a motivating influence and by clarifying questions.

A handful of meta-analyses compared the effects of guided versus unguided IBIs. A recent meta-analysis (Moshe et al., 2021) found larger effect sizes in interventions that involved human therapeutic guidance (*g* = 0.63) compared with pure self-help, i.e., unguided, interventions (*g* = 0.34), corroborating results of an older meta-analysis (Richards and Richardson, 2012). In direct comparisons, however, results are mixed (Baumeister et al., 2014; Bur, Krieger, et al., 2022; Zagorscak et al., 2018). Besides higher power in meta-analyses, another possible reason for this discrepancy could be that contact before an IBI rarely is considered in meta-analyses as an important source of variability.

### 1.4 The relative importance of contact before and during an intervention

A review by Johansson and Andersson (2012) raised the question of the relative importance of contact before and during internet-based interventions for depression. Their review considered 33 comparisons of IBIs with control groups from 25 randomized trials. Four levels of degrees of human contact were defined: 0, no contact neither before nor during the intervention; 1, contact before the intervention only, e. g., for a diagnostic interview; 2, contact during the intervention, i.e., guidance, only; and 3, contact both before and during the intervention. The authors assumed that contact during an intervention would be of greater clinical importance regarding depressive symptoms. Meta-analytic results supported their assumptions; the between-group effect sizes vis-à-vis control increased with increasing levels of contact (Cohen's *d* for degrees of contact 0 to 3 equaled 0.21, 0.44, 0.58, and 0.76, respectively). This result was also reflected in a Spearman correlation of ρ = 0.64 (*p* < .01) between the degree of support and effect size. At least three conclusions could be drawn from these results: First, contact before treatment seems beneficial. Second, contact during the intervention seemed more beneficial than contact before the intervention only. Third, interventions seemed most beneficial when contact was provided before and during the intervention. Clearly, if proven robust, these findings have important clinical implications for designing and implementing IBIs for depression. However, further replications are needed, given the relatively small number of included trials and the associated uncertainty of effect estimates. In the context of a randomized factorial trial investigating various components of IBIs for patients with increased levels of depressive symptoms via direct comparisons (Bur, Krieger, et al., 2022), results showed a somewhat more complex pattern. Regarding the above-described conclusions, results of Bur and colleagues' study showed 1) that the diagnostic interview itself did not show to have a direct effect, 2) that guidance had an effect on outcome and adherence, and 3) that the interaction between a diagnostic interview and guidance seems to be of importance, i.e., contact before the intervention in combination without contact during the intervention led to the worst outcome. Hence, the evidence of the importance of contact before the treatment seems controversial, especially whether it has an effect on its own.

### 1.5 Contact and adherence

Apart from outcome, treatment adherence, i.e., the extent to which participants use a self-help program was investigated in several studies. In general, meta-analyses have found that contact during the intervention, i.e., guidance, seems to be associated with higher adherence and lower dropout to self-help interventions for depression (Baumeister et al., 2014; Moshe et al., 2021) as well as for general mental health problems including depression (Musiat et al., 2022). Regarding the effects of contact before the intervention in depression, there is only the above-mentioned study by Bur, Krieger, et al. (2022), which did not find an effect of a diagnostic interview on adherence.

### 1.6 Aims of the present study

The present study's goal was to replicate and extend the finding by Johansson and Andersson (2012) regarding the relationship between the degree of contact and outcome in IBIs for depression. More specifically, we were interested in the independent, relative, and additive effects of contact before and during the intervention. To this end, we conducted a meta-analysis for the efficacy of IBIs for depression compared to control condition for different degrees of contact in adults suffering from elevated symptoms of depression regarding depression outcomes. In addition, intervention adherence and dropout were extracted as secondary outcomes.

## 2 Methods

A study protocol with preregistration of variable definitions, hypotheses, and analytic strategy was registered with OSF (www.osf.io/4mzyd). The reporting of the present study follows the PRISMA 2020 statement (Page et al., 2021) (for checklist, see online supplementary material).

### 2.1 Inclusion criteria

We included randomized trials that contrasted (a) one or more IBI(s) with (b) treatment as usual (TAU) or a waiting list (WL) in (c) adults with elevated symptoms of depression (including subthreshold depression, first-time and recurrent major depressive disorder, and dysthymia/persistent depressive disorder). Inclusion of a control condition was necessary because, in keeping with Johansson and Andersson (2012), our aim was to estimate IBIs effect in contrast to control. We excluded (a) trials focused on relapse prevention and (b) trials in which all patients shared a comorbid mental disorder (e.g., substance use disorder), comorbid somatic disorder (e.g., heart surgery), or comorbid social problem (e.g., unemployment). We further excluded (c) trials investigating the effects of a treatment component rather than a stand-alone treatment, (d) trials that focused on bipolar affective disorders, and (e) trials published before the year 2000. We imposed no language restrictions on reports.

### 2.2 Literature search

We (a) searched PsycINFO database for meta-analyses on psychotherapy for depression published from 2015 to September, 14th, 2021 to screen reference lists for relevant trials. Furthermore, (b) we searched PsycINFO and the Cochrane's Central Register of Controlled Trials (CENTRAL) from 2018 to September, 14th, 2021 to identify newer trials. We used search terms relating to psychotherapy, depression, and randomized trial methodology (see online Table S1 for exact search terms). Search in CENTRAL was limited to entries from EMBASE and PubMed. Search in PsycINFO was limited to entries from peer-reviewed journals. In a first step, titles and abstracts were single assessed, and in a second step, full texts were double assessed with disagreements being resolved by consensus.

### 2.3 Variable definitions and coding

Detailed coding rules for all variables were defined in a pre-registered codebook (www.osf.io/4mzyd). All information was always extracted by two independent coders. Disagreements were resolved through consensus.

#### 2.3.1 Degree of contact

Degree of CONTACT was defined as in Johansson and Andersson (2012), i.e., 0, no CONTACT neither before nor during the intervention; 1, CONTACT before intervention only, e.g., for a diagnostic interview; 2, CONTACT during the intervention, i.e., guidance, only; and 3, CONTACT both before AND during the intervention.

#### 2.3.2 Risk of bias

The revised Cochrane risk of bias tool (RoB 2) was used to assess risk of within-study bias (Sterne et al., 2019). The tool assesses risk of bias from five domains (i.e., randomization process, deviations from intended interventions, missing outcome data, outcome assessment, and selective reporting). Risk of bias from deviations from intended interventions was judged by the presence of adequate measures to ensure treatment fidelity (Munder and Barth, 2018). Use of self-report questionnaires was judged as low risk of bias from outcome assessment. Selective reporting of results was judged by comparing outcome measures that were planned a priori in study protocols with the outcome measures that were reported in trial reports. Trials fulfilling all five domains were considered at low risk of bias, all others were considered to be at some concerns or high risk of bias. Small-study effects were analyzed to assess risk of bias across trials.

#### 2.3.3 TAU intensity

Following Munder et al. (2022), we also assessed the intensity of TAU control groups, that is, the degree to which “specific or nonspecific elements of common depression treatments are provided to participants” (p. 201) in TAU. Six aspects of TAU intensity were assessed, namely, whether all participants in control groups (a) received some form of treatment for depression and (b) had unconstrained access to usual care; whether control group participants who received treatment (c) received specialized care for depression, (d) were treated by mental health professionals, (e) were treated by providers with access to training and/or supervision, and (f) received a minimum treatment dose. Based on these aspects we distinguished TAU control groups in which participants received some treatment (at least one aspect fulfilled) from TAU control groups in which participants received no treatment (no aspect fulfilled). WL was regarded as no treatment control (see also Michopoulos et al., 2021).

#### 2.3.4 Treatment outcome

The primary outcome was depressive symptoms at termination expressed as standardized mean difference (SMD). Positive SMDs indicate more change in the psychotherapy group compared to the control group. We extracted all reported patient- and observer-rated depression instruments.

#### 2.3.5 Secondary outcomes

The secondary outcome was adherence and dropout. The extracted features were the average proportion of intervention modules completed, i.e., intervention adherence, and the proportion of participants completing post-assessments, i.e., study dropout.

### 2.4 Statistical analyses

We used non-parametric methods to test for differences regarding the degree of contact. We calculated Hedges' *g* as a measure of SMD. Means and standard deviations were preferred for calculations; if unavailable, we approximated means from change scores, standard deviations from standard errors or confidence intervals, or extracted SMDs as reported in trial reports. We preferred to calculate SMDs based on intention-to-treat analyses. We used inverse-variance weighted, random-effects meta-analytic methods, as implemented in STATA's ‘meta’ command. We aggregated multiple depression instruments within trials using R package ‘MAd,’ assuming a correlation of *r* = 0.50 between instruments (Del Re and Hoyt, 2014). In trials with more than one eligible intervention, we adjusted standard errors (Higgins et al., 2021). Adherence outcomes were given as proportions and thus were arcsine-transformed for meta-analysis and back-transformed for reporting in text (Schwarzer and Rücker, 2022). We used ‘meta’ default settings which included restricted maximum likelihood estimation and significance tests based on the normal distribution. The *I*^2^ statistic was used to quantify the amount of heterogeneity between trials, with 0 % indicating no heterogeneity, and values of 25 %, 50 %, and 75 % indicating low, moderate, and strong heterogeneity, respectively. Egger's test was used to test for the presence of publication bias. SMDs ≥2 were defined as outliers. We used meta-regression to estimate the effect of the degree of CONTACT on outcome. For pre-registered meta-regressions, we introduced type of control group as the first predictor to adjust for differences between TAU and WL. Significance tests for the effect of degree of CONTACT were one-sided at alpha = 0.05, and all other tests were two-sided at alpha = 0.05.

## 3 Results

### 3.1 Included trials

We retrieved 473 references from 87 meta-analyses and 8997 references from bibliographic databases. We screened 7.746 unique references, 904 full texts and finally included 56 eligible trials (see online Fig. S1 for PRISMA flowchart). In these trials, 13,335 patients were randomized to 75 eligible psychotherapy arms (7361 patients) and 56 control arms (5916 patients). Twenty-three trials (41.07 %) used a TAU control group, and 33 trials (58.93 %) used a WL control group. In terms of control group intensity, 19 trials (33.93 %) used some treatment control group, while 37 trials (66.07 %) used a no-treatment control group. Of 75 internet-based intervention conditions included, 40 (60.00 %) provided CONTACT before treatment, while 30 (40.00 %) provided no CONTACT before treatment, or information on CONTACT before treatment was lacking. Thirty-seven (49.33 %) treatments provided CONTACT during treatment, while 38 (50.67 %) treatments provided no CONTACT during treatment, or information on CONTACT during treatment was lacking. Twelve out of 56 trials (21.43 %) were judged to have a low risk of bias according to the revised Cochrane risk of bias tool, while 44 trials (78.57 %) were judged to show some concerns or a high risk of bias (see online suppl supplementary Note S1 and Table S1 for an overview of the included trials). See online supplementary Fig. S2 for a forest plot of all 75 effects of internet-based psychotherapy versus control group (stratified for degree of CONTACT).

### 3.2 Preregistered replication of Johansson and Andersson (2012)

In preregistered meta-regressions, we looked at the relation of levels of CONTACT, as proposed by Johansson and Andersson (2012), with the effect of internet-based self-help interventions (INT) versus control, when adjusting for a possible effect of type of control group (TAU or WL) (see Table 1). In the first model across all 76 treatment comparisons, CONTACT emerged as a significant predictor (one-sided *p* < .001), with the effect of INT increasing by 0.118 for a one-level increase in CONTACT. Heterogeneity was substantial, with *I*^2^ = 67.27 %. CONTACT remained a significant predictor in two preregistered sensitivity analyses adjusting for small-study effects (by restricting meta-regression to 43 comparisons from trials with total sample sizes of ≥100 as Egger's test suggested the presence of small-study effects at *p* = .005) and the presence of risk of bias (by introducing risk of bias as a further predictor). No sensitivity analysis excluding outliers was conducted because no outlying effect (SMD > 2) was detected. A Spearman correlation of ⍴ = 0.474 (*p* < .001) also suggested a strong association between CONTACT and outcome.

**Table 1:** Preregistered meta-regressions of degree of human contact on the effect of internet-based psychotherapy for depression versus control.

Model	Variables	*k*	*B*	*SE*	*p*	*I*^2^
All comparisons	Intercept	75	0.276	0.058	0.000	67.43 %
	Type of control		−0.119	0.037	0.001	
	CONTACT		0.114	0.030	0.000^a^	
Trials with *N* ≥ 100	Intercept	43	0.280	0.062	0.000	72.88 %
	Type of control		−0.081	0.041	0.048	
	CONTACT		0.078	0.034	.011^a^	
Adjusted for risk of bias	Intercept	75	0.264	0.060	0.000	66.87 %
	Type of control		−0.117	0.038	0.002	
	Risk of bias		−0.032	0.041	0.445	
	CONTACT		0.112	0.031	0.000^a^	

a One-sided test. *k* = number of treatment contrasts. Type of control: −1 = wait list, 1 = treatment as usual. CONTACT reflects Johansson and Andersson's (2012) score of support, ranging from 0 = *no CONTACT* to 3 = *CONTACT before and during therapy*. Risk of bias: −1 = high risk of bias or some concerns, 1 = low risk of bias. Estimates from random-effects meta-analyses.

### 3.3 Exploratory analyses

#### 3.3.1 Testing the linearity of Johansson and Andersson's CONTACT score

**Fig. 1:**
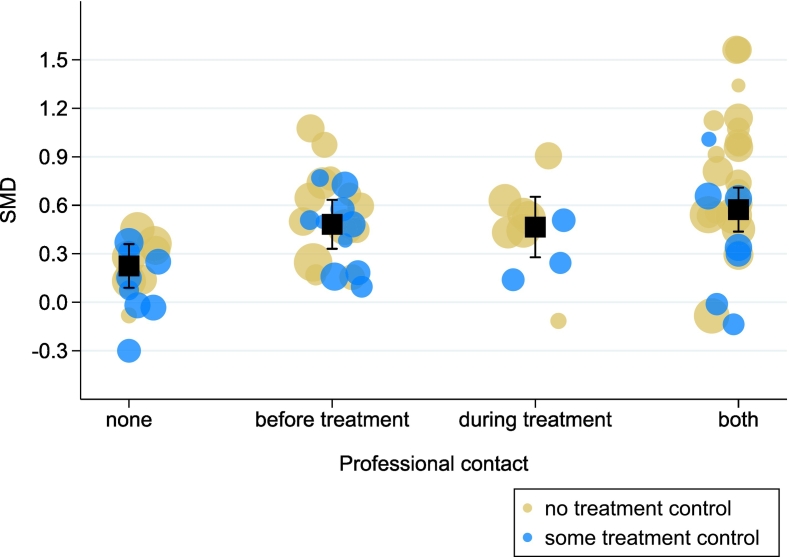
Effect of internet-based psychotherapy for depression at different levels of CONTACT. Estimates are adjusted for control group intensity (−1 = no treatment control, 1 = some treatment control). Estimates from random-effects meta-analysis. Error bars represent 95 % confidence intervals. SMD = standardized mean difference.

To further test the implicated linearity of Johansson and Andersson's (2012) CONTACT score, we calculated mean effects for each level of the score (see Fig. 1). We adjusted the meta-regression for the intensity of control groups, rather than for the type of control group, following a suggestion from one of our previous studies (Munder et al., 2022). Although there was some indication of a linear effect of CONTACT, with treatment effects being lowest without any CONTACT (SMD = 0.224, 95 % CI: 0.090, 0.340) and highest with CONTACT before and during treatment (SMD = 0.573, 95 % CI: 0.437, 0.709), there was no difference between the effects with CONTACT before treatment (SMD = 0.482, 95 % CI: 0.331, 0.633) and with CONTACT during treatment (SMD = 0.465, 95 % CI: 0.278, 0.652).

#### 3.3.2 Relation of the degree of contact with adherence and dropout

We further explored patient adherence to interventions and patient dropout from the study as secondary outcomes (see Table 2). In a meta-regression based on 45 comparisons with information on patient adherence, CONTACT emerged as a significant predictor of patient adherence, with more CONTACT being associated with higher adherence (*p* = .004), and with a patient adherence of 41.39 % for interventions without any CONTACT (SE = 14.43 %), of 62.22 % for INT with CONTACT before treatment (SE = 14.43 %), of 62.24 % for INT with CONTACT during treatment (SE = 18.90), and of 65.77 % for INT with CONTACT before and during treatment (SE = 13.36 %). In a further meta-regression based on 64 comparisons with information on patient dropout, CONTACT emerged as a significant predictor of patient dropout, with more CONTACT being associated with lower dropout (*p* = .036), with a patient dropout of 36.04 % for INT without CONTACT (SE = 12.91 %), of 16.60 % for INT with CONTACT before treatment (SE = 12.13 %), of 32.73 % for INT with CONTACT during treatment (SE = 17.68 %), and of 18.74 % for INT with CONTACT before and during treatment (SE = 10.21 %). Meta-regressions for both adherence and dropout exhibited strong heterogeneity of *I*^2^ > 90 %, suggesting strong variations in individual estimates, and both showed no clear linear effects of the CONTACT score.

**Table 2:** Degree of CONTACT as a predictor of patient adherence and patient dropout.

Outcome variable	Predictors	*k*	*B*	*SE*	*p*	*I*^2^
Patient adherence	Intercept	45	0.751	0.049	0.000	92.48 %
	CONTACT		0.074	0.026	0.004	
Patient dropout	Intercept	64	0.582	0.043	0.000	93.06 %
	CONTACT		−0.045	0.022	0.036	

Note. Patient adherence and patient dropout are represented by arcsine-transformed proportions.

#### 3.3.3 CONTACT before and during treatment separately

As the previous meta-regressions indicated that the effect of CONTACT, as conceptualized by Johansson and Andersson (2012), might not be linear, we looked at the effects of CONTACT before and CONTACT during separately. Table 3 shows the results of these analyses. Results indicate that both variables had a significant effect on treatment outcome and treatment adherence. However, regarding patient dropout, only CONTACT before the intervention had a significant effect (*p* < .001), but not CONTACT during the intervention (*p* = .464).

**Table 3:** Contact before and contact during the intervention as independent predictors of treatment outcome, patient adherence, and patient dropout.

Outcome variable	Predictors	*k*	*B*	*SE*	*p*	*I*^2^
Treatment outcome	Intercept	75	0.308	0.056	0.000	68.72 %
	Control intensity		−0.117	0.040	0.004	
	CONTACT BEFORE		0.219	0.078	0.005	
	Intercept	75	0.339	0.052	0.000	70.08 %
	Control intensity		−0.130	0.040	0.001	
	CONTACT DURING		0.188	0.078	0.016	
Patient adherence	Intercept	45	0.774	0.048	0.000	92.93 %
	CONTACT BEFORE		0.155	0.064	0.015	
	Intercept	45	0.799	0.044	0.000	93.11 %
	CONTACT DURING		0.134	0.064	0.036	
Patient dropout	Intercept	64	0.632	0.039	0.000	91.55 %
	CONTACT BEFORE		−0.196	0.049	0.000	
	Intercept	64	0.529	0.054	0.000	93.65 %
	CONTACT DURING		−0.039	0.038	0.464	

Note. Patient adherence and patient dropout are represented by arcsine-transformed proportions.

## 4 Discussion

This study set out to investigate the impact of the degree of contact on the effects of internet-based interventions for adult depression in randomized trials and thereby replicate the finding of Johansson and Andersson (2012). More specifically, we were interested in the independent, relative, and additive effects of contact before and during the intervention. In 56 trials that compared internet-based interventions with a control group, we found the degree of contact to predict intervention effects, with effects increasing with a higher degree of contact. This effect of contact was robust across a series of preregistered sensitivity analyses, including tests for the impact of small-study effects, and risk of bias. These results seemed to replicate the findings by Johansson and Andersson (2012). However, in further exploratory analyses, we found no clear evidence for the proposed linearity of the scale. While there was a marked difference in the effect on depression between interventions without any contact and interventions with contact before and during the intervention, no difference emerged between interventions with contact before intervention and intervention with contact during the intervention, which fell in-between the two former intervention types. When looking at the effects separately, both contact before and during the intervention emerged as significant predictors of outcome and adherence. Thus, our results point to a similar importance of contact before and contact during intervention. While the effects of contact during the intervention, i.e., guidance, replicate findings of earlier meta-analyses on the topic (Moshe et al., 2021; Richards and Richardson, 2012), the finding of independent effects of contact before the intervention is new and refines, in the present form, the findings by Johansson and Andersson (2012). These results indicate the incremental value and the importance of contact before the internet-based intervention. There are at least two potential explanations for this finding. First, an explanation might be methodological. A diagnostic interview ensures that only people for whom the intervention is meant for receive the treatment while other people are excluded from a study and in some cases referred to a more suitable intervention or treatment. However, in the study by Bur et al. (Bur, Krieger, et al., 2022), the outcome of the diagnostic interview had no impact on the in- or exclusion in the study and they found no incremental effect of a diagnostic interview. A second explanation could be that people perceive interventions with a human contact before the intervention as more professional and, therefore, more suited for their problem. This interpretation is in line with some aspects of the supportive accountability model by Mohr et al. (2011), which focuses on how human support affects participants' feelings of accountability. A contact before the treatment could especially impact the legitimacy of the treatment provider. Legitimacy is given by an instrumental factor (users perceive the treatment provider as having expertise and the contact with them is reciprocal). On the other hand, legitimacy is given by a relational factor (users trust the treatment provider and perceive them as benevolent). A diagnostic interview may play a crucial role, since it is, in most internet-based interventions, the only synchronous contact in person or over the phone, which, in the case of guided interventions, is followed by asynchronous contact. Future studies should look at whether the specific content of the synchronous contact before the intervention is crucial or not and whether this also holds for blended interventions, i.e., interventions that combine face-to-face and online elements.

The results of the present meta-analysis regarding contact before the intervention contrast with the results of the full factorial design study by Bur and colleagues (Bur, Krieger, et al., 2022), since in a direct comparison, a diagnostic interview did not have an impact either on outcome or adherence. On the other hand, results are in line with the effect of contact during the intervention regarding outcome. Interestingly, Bur, Krieger, et al. (2022) found that when not followed with guidance, a diagnostic interview led to the least favorable outcomes of all four possible combinations of these two factors, a finding that is incompatible with our results. One explanation for this discrepancy could be that participants in Bur et al. might have experienced what has been called resentful demoralization, i.e., they might have felt that they receive the “less desirable treatment” (Shadish et al., 2002, p. 80). However, it is important to note that in none of the trials included in this meta-analysis, the degree of contact was manipulated experimentally, and results are based not on direct comparisons. On the other hand, Bur et al. excluded patients with severe depressive symptomatology, possibly explaining the contradictory finding. This leads to the hypothesis that a diagnostic interview could not be as crucial for less heavily burdened patients, which should be tested in future studies.

Furthermore, the degree of contact emerged as a significant predictor of patient adherence, with more contact being associated with higher intervention adherence and less dropout. When looking at the effects separately, both contact before and during the intervention led to higher adherence, while contact before the intervention led to fewer dropouts. Interestingly, a recent secondary analysis of the above-mentioned factorial design study by Bur et al. (Bur, Bielinski, et al., 2022) found that both adherence and the alliance with the treatment provider independently mediated the relationship between guidance (vs. no guidance) and treatment outcome. This result indicates that contact with a human being as well as adhering to an online self-help intervention are of similar importance.

Because internet-based interventions without any contact before or during the intervention showed a small effect size (SMD = 0.224) that can even be considered not clinically relevant (Cuijpers et al., 2014), the field of application of such interventions should be well reflected on; for example, it could only be recommended in the field of prevention, for minimal depressive symptoms, or when there is a clear patient preference. This seems especially indicated since the experience of an unsuccessful treatment could lead to a (further) demoralization of patients suffering from depression and, as a consequence, negatively influence the further uptake of alternative treatment options. However, this hypothesis must be investigated in future research. Relatedly, the cost-effectiveness should be investigated and compared for the different levels of contact in future studies.

A number of limitations and possible sources of imprecision need to be discussed. Heterogeneity was rather high in all tests; this was especially true in the cases of intervention adherence and study dropout (> 90 %). This speaks for potential moderators. For example, a recent network meta-analysis (Karyotaki et al., 2021) found that both guided and unguided IBI modalities for depression outperformed control groups regardless of depression severity. However, in individuals with mild/subthreshold depression, there was little or no additional benefit from guidance, while guidance was associated with higher effects in individuals with moderate and severe depression. Furthermore, it could also be that not only patient characteristics but also characteristics of the self-help program, the fit of a program and a patient, and the quality of guidance play an important role. Possibly, guidance and quality of guidance become more important if a self-help program fits less (Berger, 2017). Future studies might need to shed more light on these moderating effects also with regard to a contact before the intervention. Another very recent meta-analysis (Koelen et al., 2022) showed that in internet treatments for any mental disorders, human guidance is more efficacious than technological guidance. In the present study, we only looked at human contact during the intervention. Relatedly, otherwise unguided interventions sometimes offer “guidance on demand”, i.e., that users can reach out to the study team if they have specific questions. We decided to code these interventions as unguided, since this form of contact does not take place regularly and is not used by all users. However, it could be that this form of potential contact has an impact on effects itself, e.g., via the supportive accountability (cf. Mohr et al., 2011), which should be tested in future studies. Additionally, we did not include trials that had no control group, i.e., we missed information from trials that compared IBIs with different degrees of contact but not with a control group. We deem it unlikely that the results of these trials differ in fundamental ways from the ones we included, however, they would have contributed to more precision. Furthermore, we did not look at the impact of human contact on follow-up time points.

Our findings add to the present literature and have implications for the importance of a synchronous contact before an internet-based intervention, as it seems crucial for contact during the intervention to have an incremental effect. To answer the question of whether this is only the case for a diagnostic interview or whether this holds for other types of pre-treatment contact as well, more research is needed.

## Declaration of competing interest

The authors declare that they have no known competing financial interests or personal relationships that could have appeared to influence the work reported in this paper.

## Appendix A Supplementary material

Supplementary material Image 1
