# Supplementary material for: Human contact in internet-based interventions for depression: A pre-registered replication and meta-analysis of randomized trials
Source: Internet Interv. 2023 Mar 31;32:100617. doi: 10.1016/j.invent.2023.100617 (PMC10235426; doi:10.1016/j.invent.2023.100617)

**Supplementary Material**

**Table of contents**

PRISMA 2020 Checklist 2

Table S1. Search terms 5

Figure S1. PRISMA flowchart 6

Note S1. References to reports on included trials 7

Table S2. Included trials contrasting internet-based psychotherapy for depression with control groups 11

Figure S2. Forest plot of effects of internet-based psychotherapy versus control group stratified for degree of CONTACT. 16

## PRISMA 2020 Checklist

| **Section and Topic** | **Item #** | **Checklist item** | **Location where item is reported** |
| --- | --- | --- | --- |
| **TITLE** | | |  |
| Title | 1 | Identify the report as a systematic review. | 1 |
| **ABSTRACT** | | |  |
| Abstract | 2 | See the PRISMA 2020 for Abstracts checklist. |  |
| **INTRODUCTION** | | |  |
| Rationale | 3 | Describe the rationale for the review in the context of existing knowledge. | 4-8 |
| Objectives | 4 | Provide an explicit statement of the objective(s) or question(s) the review addresses. | 8 |
| **METHODS** | | |  |
| Eligibility criteria | 5 | Specify the inclusion and exclusion criteria for the review and how studies were grouped for the syntheses. | 8-9 |
| Information sources | 6 | Specify all databases, registers, websites, organisations, reference lists and other sources searched or consulted to identify studies. Specify the date when each source was last searched or consulted. | 9 |
| Search strategy | 7 | Present the full search strategies for all databases, registers and websites, including any filters and limits used. | Note S1 |
| Selection process | 8 | Specify the methods used to decide whether a study met the inclusion criteria of the review, including how many reviewers screened each record and each report retrieved, whether they worked independently, and if applicable, details of automation tools used in the process. | 11 & Fig. S1 |
| Data collection process | 9 | Specify the methods used to collect data from reports, including how many reviewers collected data from each report, whether they worked independently, any processes for obtaining or confirming data from study investigators, and if applicable, details of automation tools used in the process. | 9 |
| Data items | 10a | List and define all outcomes for which data were sought. Specify whether all results that were compatible with each outcome domain in each study were sought (e.g. for all measures, time points, analyses), and if not, the methods used to decide which results to collect. | 9, Note S1, coding sheet |
|  | 10b | List and define all other variables for which data were sought (e.g. participant and intervention characteristics, funding sources). Describe any assumptions made about any missing or unclear information. | 9, Note S1, coding sheet |
| Study risk of bias assessment | 11 | Specify the methods used to assess risk of bias in the included studies, including details of the tool(s) used, how many reviewers assessed each study and whether they worked independently, and if applicable, details of automation tools used in the process. | Note S2 |
| Effect measures | 12 | Specify for each outcome the effect measure(s) (e.g. risk ratio, mean difference) used in the synthesis or presentation of results. | 10 |
| Synthesis methods | 13a | Describe the processes used to decide which studies were eligible for each synthesis (e.g. tabulating the study intervention characteristics and comparing against the planned groups for each synthesis (item #5)). | Not applicable |
|  | 13b | Describe any methods required to prepare the data for presentation or synthesis, such as handling of missing summary statistics, or data conversions. | 11 |
|  | 13c | Describe any methods used to tabulate or visually display results of individual studies and syntheses. | Figure S2 |
|  | 13d | Describe any methods used to synthesize results and provide a rationale for the choice(s). If meta-analysis was performed, describe the model(s), method(s) to identify the presence and extent of statistical heterogeneity, and software package(s) used. | 11 |
|  | 13e | Describe any methods used to explore possible causes of heterogeneity among study results (e.g. subgroup analysis, meta-regression). | 11 |
|  | 13f | Describe any sensitivity analyses conducted to assess robustness of the synthesized results. | 12 |
| Reporting bias assessment | 14 | Describe any methods used to assess risk of bias due to missing results in a synthesis (arising from reporting biases). | 12 |
| Certainty assessment | 15 | Describe any methods used to assess certainty (or confidence) in the body of evidence for an outcome. | Not conducted |
| **RESULTS** | | |  |
| Study selection | 16a | Describe the results of the search and selection process, from the number of records identified in the search to the number of studies included in the review, ideally using a flow diagram. | 11, Figure S1 |
|  | 16b | Cite studies that might appear to meet the inclusion criteria, but which were excluded, and explain why they were excluded. | Not applicable |
| Study characteristics | 17 | Cite each included study and present its characteristics. | Note S1 |
| Risk of bias in studies | 18 | Present assessments of risk of bias for each included study. | Table S1 |
| Results of individual studies | 19 | For all outcomes, present, for each study: (a) summary statistics for each group (where appropriate) and (b) an effect estimate and its precision (e.g. confidence/credible interval), ideally using structured tables or plots. | Figure S2 |
| Results of syntheses | 20a | For each synthesis, briefly summarise the characteristics and risk of bias among contributing studies. | 12, Table 1 |
|  | 20b | Present results of all statistical syntheses conducted. If meta-analysis was done, present for each the summary estimate and its precision (e.g. confidence/credible interval) and measures of statistical heterogeneity. If comparing groups, describe the direction of the effect. | 12-16 |
|  | 20c | Present results of all investigations of possible causes of heterogeneity among study results. | Table 1 |
|  | 20d | Present results of all sensitivity analyses conducted to assess the robustness of the synthesized results. | Table 1 |
| Reporting biases | 21 | Present assessments of risk of bias due to missing results (arising from reporting biases) for each synthesis assessed. | 12 |
| Certainty of evidence | 22 | Present assessments of certainty (or confidence) in the body of evidence for each outcome assessed. | Not conducted |
| **DISCUSSION** | | |  |
| Discussion | 23a | Provide a general interpretation of the results in the context of other evidence. | 16-19 |
|  | 23b | Discuss any limitations of the evidence included in the review. | 19 |
|  | 23c | Discuss any limitations of the review processes used. | 19 |
|  | 23d | Discuss implications of the results for practice, policy, and future research. | 17-19, 20 |
| **OTHER INFORMATION** | | |  |
| Registration and protocol | 24a | Provide registration information for the review, including register name and registration number, or state that the review was not registered. | 9 |
|  | 24b | Indicate where the review protocol can be accessed, or state that a protocol was not prepared. | 9 |
|  | 24c | Describe and explain any amendments to information provided at registration or in the protocol. | NA |
| Support | 25 | Describe sources of financial or non-financial support for the review, and the role of the funders or sponsors in the review. | No funding |
| Competing interests | 26 | Declare any competing interests of review authors. | No COI |
| Availability of data, code and other materials | 27 | Report which of the following are publicly available and where they can be found: template data collection forms; data extracted from included studies; data used for all analyses; analytic code; any other materials used in the review. | Following publication, the full matrix of data used in this meta-analysis will be made available at OSF. |

From: Page, M. J., McKenzie, J. E., Bossuyt, P. M., Boutron, I., Hoffmann, T. C., Mulrow, C. D., . . . Moher, D. (2021). The PRISMA 2020 statement: an updated guideline for reporting systematic reviews. BMJ, 372, n71. doi:10.1136/bmj.n71

## Table S1. Search terms

| **Identification of RCTs in CENTRAL and PsycINFO** |
| --- |
| Randomized controlled trial (applied in All Text):  random* or ((control* or compar* or clin*) N3 (trial* or studi* or study))    Psychotherapy (applied in Title, Abstract, and Keywords):  (Psychotherap* or ((acceptance or attachment or behavio#r* or analytic* or brief or cognitive or dynamic or psychodynamic or emotion* or feminist or sensiti?ation or existen* or exposure or gestalt or humanistic or hypno* or integr* or short-term* or shortterm* or long-term* or longterm* or interpersonal* or inter-personal* or mindfulness* or multimodal or narrative or positive or psychol* or rational* or solution* or eclectic* or experiential* or expressive* or insight* or supportive* or schema*) N5 (treatment* or therapy or therapies or psychotherapy or intervention*))) or (((web or digital or Internet or online or computer or app* or application* or smartphone or smart-phone) N5 (therapy or therapies or psychotherapy or intervention* or treatment*)) or ((guided or unguided or assisted or therapist*) N5 self*) or e-therapy or icbt)  Depression (applied in Title and Keywords):  depression or depressive or dysthymi* |
| **Identification of systematic reviews in PsycINFO** |
| Systematic review methodology (applied in Title):   meta-analy* or (systematic review)    Psychotherapy and Depression sections were identical to the randomized trials search described above. |

Search terms given in EBSCO syntax for PsycINFO. CENTRAL = Cochrane Central Register of Controlled Trials

##

## Figure S1. PRISMA flowchart

8,997 records identified by database search

4,828 from CENTRAL

4,169 from PsycINFO

473 records from 87 systematic reviews

7,746 records after removing duplicates

6,842 records excluded

7,746 records screened

848 trial reports excluded

150 no internet-based therapy

118 control group not treatment as usual or waiting list

88 not all patients depressed

13 not treatment-seeking adults

39 no randomized design

14 no acute depression

111 comorbid mental or somatic disorder

144 comorbid social problem or specific sociodemographic group

5 component study

1 no peer review

54 published before 2000

88 study protocol or secondary publication

22 no depression outcome reported

1 no separate outcome for type of internet-based therapy

904 trial reports reviewed in depth

56 eligible trials

## Note S1. References to reports on included trials

Addington EL, Cheung EO, Bassett SM, Kwok I, Schuette SA, Shiu E, et al. The MARIGOLD study: Feasibility and enhancement of an online intervention to improve emotion regulation in people with elevated depressive symptoms. J Affect Disord. 2019 Oct;257:352-64.

Andersson G, Bergström J, Holländare F, Carlbring P, Kaldo V, Ekselius L. Internet-based self-help for depression: randomised controlled trial. British J Psychiatry. 2005 Nov;187(5):456-61.

Beevers CG, Pearson R, Hoffman JS, Foulser AA, Shumake J, Meyer B. Effectiveness of an internet intervention (Deprexis) for depression in a United States adult sample: A parallel-group pragmatic randomized controlled trial. J Consult Clin Psychol. 2017 Apr;85(4):367-80.

Berger T, Hämmerli K, Gubser N, Andersson G, Caspar F. Internet-based treatment of depression: a randomized controlled trial comparing guided with unguided self-help. Cogn Behav Ther. 2011 Nov;40(4):251-66.

Bolier L, Haverman M, Kramer J, Westerhof GJ, Riper H, Walburg JA, et al. An Internet-based intervention to promote mental fitness for mildly depressed adults: randomized controlled trial. J Med Internet Res. 2013 Sep;15(9):e200.

Bücker L, Schnakenberg P, Karyotaki E, Moritz S, Westermann S. Diminishing effects after recurrent use of self-guided internet-based interventions in depression: randomized controlled trial. J Med Internet Res. 2019 Oct;21(10):e14240.

Buntrock C, Ebert D, Lehr D, Riper H, Smit F, Cuijpers P, et al. Effectiveness of a web-based cognitive behavioural intervention for subthreshold depression: pragmatic randomised controlled trial. Psychother Psychosom. 2015 Sep;84(6):348-58.

Carlbring P, Hägglund M, Luthström A, Dahlin M, Kadowaki Å, Vernmark K, et al. Internet-based behavioral activation and acceptance-based treatment for depression: a randomized controlled trial. J Affect Disord. 2013 Jun;148(2-3):331-7.

Clarke G, Eubanks D, Reid E, Kelleher C, O'Connor E, DeBar LL, et al. Overcoming depression on the internet (ODIN)(2): a randomized trial of a self-help depression skills program with reminders. J Med Internet Res. 2005 Apr-Jun;7(2):e16.

Clarke G, Reid E, Eubanks D, O'connor E, DeBar LL, Kelleher C, et al. Overcoming depression on the Internet (ODIN): a randomized controlled trial of an Internet depression skills intervention program. J Med Internet Res. 2002 Dec;4(3):e14.

Dahne J, Lejuez C, Diaz VA, Player MS, Kustanowitz J, Felton JW, et al. Pilot randomized trial of a selfhelp behavioral activation mobile app for utilization in primary care. Behav Ther. 2019 Jul;50(4):817-27.

De Graaf L, Gerhards S, Arntz A, Riper H, Metsemakers J, Evers S, et al. Clinical effectiveness of online computerised cognitive–behavioural therapy without support for depression in primary care: randomised trial. Brit J Psychiatry. 2009 Jul;195(1):73-80.

Ebert DD, Buntrock C, Lehr D, Smit F, Riper H, Baumeister H, et al. Effectiveness of web-and mobilebased treatment of subthreshold depression with adherence-focused guidance: a single-blind randomized controlled trial. Behav Ther. 2018 Jan;49(1):71-83.

Everitt N, Broadbent J, Richardson B, Smyth JM, Heron K, Teague S, et al. Exploring the features of an app-based just-in-time intervention for depression. J Affect Disord. 2021 May;291:279-87.

Farrer L, Christensen H, Griffiths KM, Mackinnon A. Internet-based CBT for depression with and without telephone tracking in a national helpline: randomised controlled trial. PLoS One. 2011 Nov;6(11):e28099.

Forand NR, Barnett JG, Strunk DR, Hindiyeh MU, Feinberg JE, Keefe JR. Efficacy of guided iCBT for depression and mediation of change by cognitive skill acquisition. Behav Ther. 2018 Mar;49(2):295-307.

Fuhr K, Fahse B, Hautzinger M, Gulewitsch MD. Implementation of an internet-based self-help for patients waiting for outpatient psychotherapy: first results. Psychother Psychosom Med Psychol. 2018 Jun;68(6):234-41.

Geraedts AS, Kleiboer AM, Wiezer NM, van Mechelen W, Cuijpers P. Short-term effects of a webbased guided self-help intervention for employees with depressive symptoms: randomized controlled trial. J Med Internet Res. 2014 May;16(5):e121.

Gilbody S, Littlewood E, Hewitt C, Brierley G, Tharmanathan P, Araya R, et al. Computerised cognitive behaviour therapy (cCBT) as treatment for depression in primary care (REEACT trial): large scale pragmatic randomised controlled trial. BMJ. 2015 Nov;351:h5627.

Gräfe V, Moritz S, Greiner W. Health economic evaluation of an internet intervention for depression (deprexis), a randomized controlled trial. Health economics review. 2020 Jun;10(1):1-11.

Hallgren M, Kraepelien M, Lindefors N, Zeebari Z, Kaldo V, Forsell Y. Physical exercise and internetbased cognitive-behavioural therapy in the treatment of depression: randomised controlled trial. Brit J Psychiatry. 2015 Sep;207(3):227-34.

Jelinek L, Arlt S, Moritz S, Schröder J, Westermann S, Cludius B. Brief web-based intervention for depression: randomized controlled trial on behavioral activation. J Med Internet Res. 2020 Mar;22(3):e15312.

Johansson O, Bjärehed J, Andersson G, Carlbring P, Lundh L-G. Effectiveness of guided internetdelivered cognitive behavior therapy for depression in routine psychiatry: a randomized controlled trial. Internet Interv. 2019 May;17:100247.

Kenter RMF, Cuijpers P, Beekman A, van Straten A. Effectiveness of a web-based guided self-help intervention for outpatients with a depressive disorder: short-term results from a randomized controlled trial. J Med Internet Res. 2016 Mar;18(3):e80.

Kivi M, Eriksson MC, Hange D, Petersson E-L, Vernmark K, Johansson B, et al. Internet-based therapy for mild to moderate depression in Swedish primary care: short term results from the PRIMNET randomized controlled trial. Cogn Behav Ther. 2014 Oct;43(4):289-98.

Kleiboer A, Donker T, Seekles W, van Straten A, Riper H, Cuijpers P. A randomized controlled trial on the role of support in Internet-based problem solving therapy for depression and anxiety. Behav Res Ther. 2015 Sep;72:63-71.

Krämer LV, Gruenzig S-D, Baumeister H, Ebert DD, Bengel J. Effectiveness of a guided web-based intervention to reduce depressive symptoms before outpatient psychotherapy: A pragmatic randomized controlled trial. Psychother Psychosom. 2021 May;90(4):233-42.

Lambert JD, Greaves CJ, Farrand P, Price L, Haase AM, Taylor AH. Web-based intervention using behavioral activation and physical activity for adults with depression (the eMotion study): pilot randomized controlled trial. J Med Internet Res. 2018 Jul;20(7):e10112.

Lappalainen P, Langrial S, Oinas-Kukkonen H, Tolvanen A, Lappalainen R. Web-based acceptance and commitment therapy for depressive symptoms with minimal support: a randomized controlled trial. Behav Modif. 2015 Nov;39(6):805-34.

Lemma A, Fonagy P. Feasibility study of a psychodynamic online group intervention for depression. Psychoanal Psychol. 2013 Jul;30(3):367-80.

MacLean S, Corsi DJ, Litchfield S, Kucharski J, Genise K, Selaman Z, et al. Coach-facilitated web-based therapy compared with information about web-based resources in patients referred to secondary mental health care for depression: randomized controlled trial. J Med Internet Res. 2020 Jun;22(6):e15001.

Löbner M, Pabst A, Stein J, Dorow M, Matschinger H, Luppa M, et al. Computerized cognitive behavior therapy for patients with mild to moderately severe depression in primary care: A pragmatic cluster randomized controlled trial (@ ktiv). J Affect Disord. 2018 Oct;238:317-26.

Lüdtke T, Pult LK, Schröder J, Moritz S, Bücker L. A randomized controlled trial on a smartphone selfhelp application (Be Good to Yourself) to reduce depressive symptoms. Psychiatry Res. 2018 Nov;269:753-62.

Meyer B, Berger T, Caspar F, Beevers C, Andersson G, Weiss M. Effectiveness of a novel integrative online treatment for depression (Deprexis): randomized controlled trial. J Med Internet Res. 2009 Apr-Jun;11(2):e15.

Meyer B, Bierbrodt J, Schröder J, Berger T, Beevers CG, Weiss M, et al. Effects of an Internet intervention (Deprexis) on severe depression symptoms: randomized controlled trial. Internet Interv. 2015 Mar;2(1):48-59.

Mira A, Bretón-López J, García-Palacios A, Quero S, Baños RM, Botella C. An Internet-based program for depressive symptoms using human and automated support: a randomized controlled trial. Neuropsychiatr Dis Treat. 2017 Mar;13:987-1006.

Mohr DC, Duffecy J, Ho J, Kwasny M, Cai X, Burns MN, et al. A randomized controlled trial evaluating a manualized TeleCoaching protocol for improving adherence to a web-based intervention for the treatment of depression. PLoS One. 2013 Aug;8(8):e70086.

Montero-Marín J, Araya R, Pérez-Yus MC, Mayoral F, Gili M, Botella C, et al. An internet-based intervention for depression in primary care in Spain: a randomized controlled trial. J Med Internet Res. 2016 Aug;18(8):e231.

Moritz S, Schilling L, Hauschildt M, Schröder J, Treszl A. A randomized controlled trial of internetbased therapy in depression. Behav Res Ther. 2012 Aug;50(7-8):513-21.

Nyström MB, Stenling A, Sjöström E, Neely G, Lindner P, Hassmén P, et al. Behavioral activation versus physical activity via the internet: A randomized controlled trial. J Affect Disord. 2017 jun;215:85-93.

Perini S, Titov N, Andrews G. Clinician-assisted Internet-based treatment is effective for depression: randomized controlled trial. Aust N Z J Psychiatry. 2009 Jun;43(6):571-8.

Pots WT, Fledderus M, Meulenbeek PA, Peter M, Schreurs KM, Bohlmeijer ET. Acceptance and commitment therapy as a web-based intervention for depressive symptoms: randomised controlled trial. British J Psychiatry. 2016 Jan;208(1):69-77.

Richards D, Timulak L, O'Brien E, Hayes C, Vigano N, Sharry J, et al. A randomized controlled trial of an internet-delivered treatment: its potential as a low-intensity community intervention for adults with symptoms of depression. Behav Res Ther. 2015 Dec;75:20-31.

Roepke AM, Jaffee SR, Riffle OM, McGonigal J, Broome R, Maxwell B. Randomized controlled trial of SuperBetter, a smartphone-based/internet-based self-help tool to reduce depressive symptoms. Games Health J. 2015 Jun;4(3):235-46.

Rosso IM, Killgore WD, Olson EA, Webb CA, Fukunaga R, Auerbach RP, et al. Internet‐based cognitive behavior therapy for major depressive disorder: A randomized controlled trial. Depress Anxiety. 2017 Mar;34(3):236-45.

Ruwaard J, Schrieken B, Schrijver M, Broeksteeg J, Dekker J, Vermeulen H, et al. Standardized webbased cognitive behavioural therapy of mild to moderate depression: a randomized controlled trial with a long-term follow-up. Cogn Behav Ther. 2009 Feb;38(4):206-21.

Schure MB, Lindow JC, Greist JH, Nakonezny PA, Bailey SJ, Bryan WL, et al. Use of a fully automated internet-based cognitive behavior therapy intervention in a community population of adults with depression symptoms: randomized controlled trial. J Med Internet Res. 2019 Nov;21(11):e14754.

Segal ZV, Dimidjian S, Beck A, Boggs JM, Vanderkruik R, Metcalf CA, et al. Outcomes of online mindfulness-based cognitive therapy for patients with residual depressive symptoms: a randomized clinical trial. JAMA psychiatry. 2020 Jan;77(6):563-73.

Smith J, Newby JM, Burston N, Murphy MJ, Michael S, Mackenzie A, et al. Help from home for depression: A randomised controlled trial comparing internet-delivered cognitive behaviour therapy with bibliotherapy for depression. Internet Interv. 2017 May;9:25-37.

Stiles-Shields C, Montague E, Kwasny MJ, Mohr DC. Behavioral and cognitive intervention strategies delivered via coached apps for depression: pilot trial. Psychol Serv. 2019 May;16(2):233-8.

Titov N, Andrews G, Davies M, McIntyre K, Robinson E, Solley K. Internet treatment for depression: a randomized controlled trial comparing clinician vs. technician assistance. PLoS One. 2010 Jun;5(6):e10939.

Tulbure BT, Andersson G, Sălăgean N, Pearce M, Koenig HG. Religious versus conventional internetbased cognitive behavioral therapy for depression. J Relig Health. 2018 Oct;57(5):1634-48.

Vernmark K, Lenndin J, Bjärehed J, Carlsson M, Karlsson J, Öberg J, et al. Internet administered guided self-help versus individualized e-mail therapy: A randomized trial of two versions of CBT for major depression. Behav Res Ther. 2010 May;48(5):368-76.

Warmerdam L, van Straten A, Twisk J, Riper H, Cuijpers P. Internet-based treatment for adults with depressive symptoms: randomized controlled trial. J Med Internet Res. 2008 Nov;10(4):e44.

Williams AD, Blackwell SE, Mackenzie A, Holmes EA, Andrews G. Combining imagination and reason in the treatment of depression: a randomized controlled trial of internet-based cognitive-bias modification and internet-CBT for depression. J Consult Clin Psychol. 2013 Oct;81(5):793-9.

Yeung A, Wang F, Feng F, Zhang J, Cooper A, Hong L, et al. Outcomes of an online computerized cognitive behavioral treatment program for treating chinese patients with depression: A pilot study. Asian J Psychiatr. 2018 Dec;38:102-7.

## Table S2. Included trials contrasting internet-based psychotherapy for depression with control groups

|  | Intervention | | |  | Control | | |  |  |
| --- | --- | --- | --- | --- | --- | --- | --- | --- | --- |
| Study | Treatment | Modality | *N* |  | Type | Intensity^a^ | *N* | Risk of bias | Outcome measure |
| Addington et al. 2019 | Positive emotion skills intervention | Self-guided INT | 26 |  | WL | lower | 15 | some concern or high | PHQ-8, CES-D |
| Andersson et al. 2005 | iCBT | Guided INT | 57 |  | WL | lower | 60 | some concern or high | BDI-I, MADRS |
| Beevers et al. 2017 | Deprexis | Self-guided INT | 285 |  | TAU/WL | higher | 91 | low | QIDS-SR, HRSD |
| Berger et al. 2011 | Deprexis (with support) | Guided INT | 25 |  | WL | lower | 26 | some concern or high | BDI-II |
|  | Deprexis (without support) | Self-guided INT | 25 |  |  |  |  |  |  |
| Bolier et al. 2013 | Psyfit | Self-guided INT | 143 |  | WL | higher | 141 | some concern or high | CES-D |
| Bücker et al. 2019 | Mood | Self-guided INT | 47 |  | TAU | higher | 56 | some concern or high | BDI-II |
|  |  |  |  |  |  |  |  |  | PHQ-9 |
| Buntrock et al. 2015 | GET.ON mood enhancer | Guided INT | 202 |  | TAU | lower | 204 | some concern or high | CES-D |
| Carlbring et al. 2013 | BA + ACT | Guided INT | 40 |  | WL | lower | 40 | some concern or high | MADRS |
|  |  |  |  |  |  |  |  |  | BDI-II |
| Clarke et al. 2002 | Depression skills program | Self-guided INT | 107 |  | TAU | higher | 116 | some concern or high | CES-D |
| Clarke et al. 2005 | Depression skills program (with postcard reminders) | Self-guided INT | 54 |  | TAU | higher | 79 | some concern or high | CES-D |
|  | Depression skills program (with telephone reminders) | Self-guided INT | 67 |  |  |  |  |  |  |
| Dahne et al. 2019 | Moodivate | Self-guided INT | 22 |  | TAU | lower | 9 | some concern or high | BDI-II |
|  | Moodkit | Self-guided INT | 18 |  |  |  |  |  |  |
| De Graaf et al. 2009 | iCBT | Self-guided INT | 95 |  | TAU | higher | 96 | low | BDI-II |
| Ebert et al. 2018 | iCBT | Guided INT | 102 |  | TAU/WL | lower | 102 | some concern or high | QIDS-SR, HRSD, CES-D |
| Everitt et al. 2021 | ImproveYourMood | Self-guided INT | 62 |  | WL | lower | 55 | some concern or high | PHQ-9 |
|  | ImproveYourMood+ | Self-guided INT | 60 |  |  |  |  |  |  |
| Farrer et al. 2011 | iCBT (with telephone tracking) | Guided INT | 18 |  | TAU | lower | 27 | some concern or high | CES-D |
|  | iCBT (without telephone tracking) | Self-guided INT | 27 |  |  |  |  |  |  |
| Forand et al. 2018 | iCBT | Guided INT | 45 |  | WL | lower | 27 | some concern or high | PHQ-9, HRSD |
| Fuhr et al. 2018 | Deprexis | Self-guided INT | 14 |  | WL | lower | 13 | some concern or high | PHQ-9 |
| Geraedts et al. 2014 | Happy@work | Guided INT | 116 |  | TAU | higher | 115 | some concern or high | CES-D |
| Gilbody et al. 2015 | Beating the Blues | Self-guided INT | 165 |  | TAU | higher | 179 | low | PHQ-9 |
|  | MoodGym | Self-guided INT | 182 |  |  |  |  |  |  |
| Graefe et al. 2020 | Deprexis | Self-guided INT | 1904 |  | TAU | higher | 1901 | some concern or high | PHQ-9 |
| Hallgren et al. 2015 | iCBT | Guided INT | 259 |  | TAU | higher | 232 | some concern or high | MADRS |
| Jelinek et al. 2020 | iBA | Self-guided INT | 29 |  | TAU | higher | 32 |  | PHQ-9 |
| Johansson et al. 2019 | iCBT | Guided INT | 27 |  | WL | lower | 27 | some concern or high | MADRS, HADS-D |
| Kenter et al. 2016 | PST | Guided INT | 136 |  | WL | lower | 133 | some concern or high | CES-D |
| Kivi et al. 2014 | iCBT | Guided INT | 30 |  | TAU | higher | 35 | some concern or high | BDI-II, MADRS |
| Kleiboer et al. 2015 | PST (without guidance) | Self-guided INT | 107 |  | WL | lower | 106 | low | CES-D |
|  | PST (with guidance on request) | Guided INT | 108 |  |  |  |  |  | PHQ-9 |
|  | PST (with weekly guidance) | Guided INT | 106 |  |  |  |  |  |  |
| Krämer et al. 2021 | WBI | Guided INT | 67 |  | TAU | higher | 69 | low | CES-D,  PHQ-9 |
| Lambert et al. 2018 | BA + physical activity | Self-guided INT | 25 |  | WL | higher | 25 | some concern or high | PHQ-8 |
| Lappalainen et al. 2015 | ACT | Guided INT | 18 |  | WL | lower | 20 | some concern or high | BDI-II |
| Lemma et al. 2013 | DIT (with therapist facilitation) | Guided INT | 8 |  | WL | lower | 8 | some concern or high | PHQ-9 |
|  | DIT (without therapist facilitation) | Self-guided INT | 8 |  |  |  |  |  |  |
| MacLean et al. 2020 | The Journal | Guided INT | 47 |  | TAU | higher | 46 | low | PHQ-9 |
| Löbner et al. 2018 | iCBT | Self-guided INT | 320 |  | TAU | lower | 327 | low | BDI-II, PHQ-9 |
| Lüdtke et al. 2018 | Be good to yourself | Self-guided INT | 44 |  | WL | lower | 44 | some concern or high | PHQ-9 |
| Meyer et al. 2009 | Deprexis | Self-guided INT | 320 |  | TAU/WL | lower | 76 | some concern or high | BDI-I |
| Meyer et al. 2015 | Deprexis | Self-guided INT | 78 |  | TAU/WL | higher | 85 | some concern or high | PHQ-9 |
| Mira et al. 2017 | Smiling is fun (automated support) | Self-guided INT | 36 |  | WL | lower | 44 | some concern or high | BDI-II, ODSIS |
|  | Smiling is fun (human support) | Guided INT | 44 |  |  |  |  |  |  |
| Mohr et al. 2013 | Mood manager (with telephone support) | Guided INT | 34 |  | WL | lower | 33 | some concern or high | PHQ-9 |
|  | Mood manager (without telephone support) | Self-guided INT | 35 |  |  |  |  |  |  |
| Montero-Marin et al. 2016 | Smiling is Fun (therapist-guided) | Guided INT | 64 |  | TAU | higher | 67 | some concern or high | BDI-II |
|  | Smiling is Fun (self-guided) | Self-guided INT | 57 |  |  |  |  |  |  |
| Moritz et al. 2012 | Deprexis | Self-guided INT | 105 |  | WL | lower | 105 | some concern or high | BDI-I |
| Nyström et al. 2017 | BA based on Lewisohn | Guided INT | 63 |  | WL | lower | 53 | some concern or high | PHQ-9 |
|  | BA based on Martell | Guided INT | 49 |  |  |  |  |  |  |
| Perini et al. 2009 | Sadness program | Guided INT | 27 |  | WL | lower | 18 | some concern or high | PHQ-9, BDI-II |
| Pots et al. 2016 | ACT | Guided INT | 82 |  | WL | lower | 87 | some concern or high | CES-D |
| Richards et al. 2015 | iCBT | Guided INT | 96 |  | WL | lower | 92 | low | BDI-II |
| Roepke et al. 2015 | SuperBetter (version with CBT and PPT) | Self-guided INT | 20 |  | WL | higher | 36 | some concern or high | CES-D |
|  | SuperBetter (general version) | Self-guided INT | 18 |  |  |  |  |  |  |
| Rosso et al. 2017 | iCBT | Self-guided INT | 37 |  | WL | lower | 40 | low | HRSD, PHQ-9 |
| Ruwaard et al. 2009 | iCBT | Guided INT | 36 |  | WL | lower | 18 | some concern or high | BDI-I, SCL-90-D |
| Schure et al. 2019 | iCBT | Self-guided INT | 181 |  | WL | higher | 162 | some concern or high | PHQ-9 |
| Segal et al. 2020 | Mindful Mood Balance | Guided INT | 230 |  | TAU | lower | 230 | low | PHQ-9 |
| Smith et al. 2017 | iCBT | Self-guided INT | 61 |  | WL | lower | 68 | low | PHQ-9 |
| Stiles-Shields et al. 2019 | BT | Guided INT | 10 |  | WL | lower | 10 | some concern or high | PHQ-9 |
|  | CT | Guided INT | 7 |  |  |  |  |  |  |
| Titov et al. 2010 | iCBT (technician-assisted) | Self-guided INT | 41 |  | WL | lower | 40 | some concern or high | PHQ-9, BDI-II |
| Tulbure et al. 2018 | iCBT (conventional) | Guided INT | 34 |  | WL | lower | 26 | some concern or high | BDI-II, IDS |
|  | iCBT (religious) | Guided INT | 19 |  |  |  |  |  |  |
| Vernmark et al. 2010 | iCBT (individualized email therapy) | Guided INT | 30 |  | WL | lower | 29 | some concern or high | BDI-I, MADRS |
|  | iCBT (guided self-help) | Guided INT | 29 |  |  |  |  |  |  |
| Warmerdam et al. 2008 | iCBT | Guided INT | 88 |  | WL | lower | 87 | low | CES-D |
|  | PST | Guided INT | 87 |  |  |  |  |  |  |
| Williams AD et al. 2013 | iCBT | Self-guided INT | 20 |  | WL | lower | 22 | some concern or high | BDI-II, PHQ-9 |
| Yeung et al. 2018 | MoodGym | Self-guided INT | 29 |  | TAU | lower | 32 | some concern or high | CES-D |

^a^Trials with lower intensity fulfilled none of the indicators of control group intensity, trials with higher intensity fulfilled ≥ 1 indicator(s). ACT = acceptance and commitment therapy; BA = behavioral activation; BDI-I = Beck Depression Inventory; BDI-II = Beck Depression Inventory revised; BT = behavioral therapy; CBT = cognitive-behavior therapy; CES-D = Center for Epidemiological Studies Depression scale; CT = cognitive therapy; DIT = dynamic-interpersonal therapy; HADS-D = Hospital Depression and Anxiety Scale - depression subscale; HRSD = Hamilton Rating Scale for Depression; iBA = internet-based behavioral activation; iCBT = internet-based cognitive-behavioral therapy; IDS = Inventory for Depressive Symptomatology; INT = internet-based psychotherapy; MADRS = Montgomery-Åsberg Depression Rating Scale; ODSIS = Overall Depression Severity and Impairment Scale; PDT = psychodynamic therapy; PHQ-8, PHQ-9 = Patient Health Questionnaire; PST = problem-solving therapy; QIDS-SR = Quick Inventory of Depressive Symptomatology; SCL-90-D = Symptom Check List-90 Depression subscale; WBI = web-based intervention.

## Figure S2. Forest plot of effects of internet-based psychotherapy versus control group stratified for degree of CONTACT.


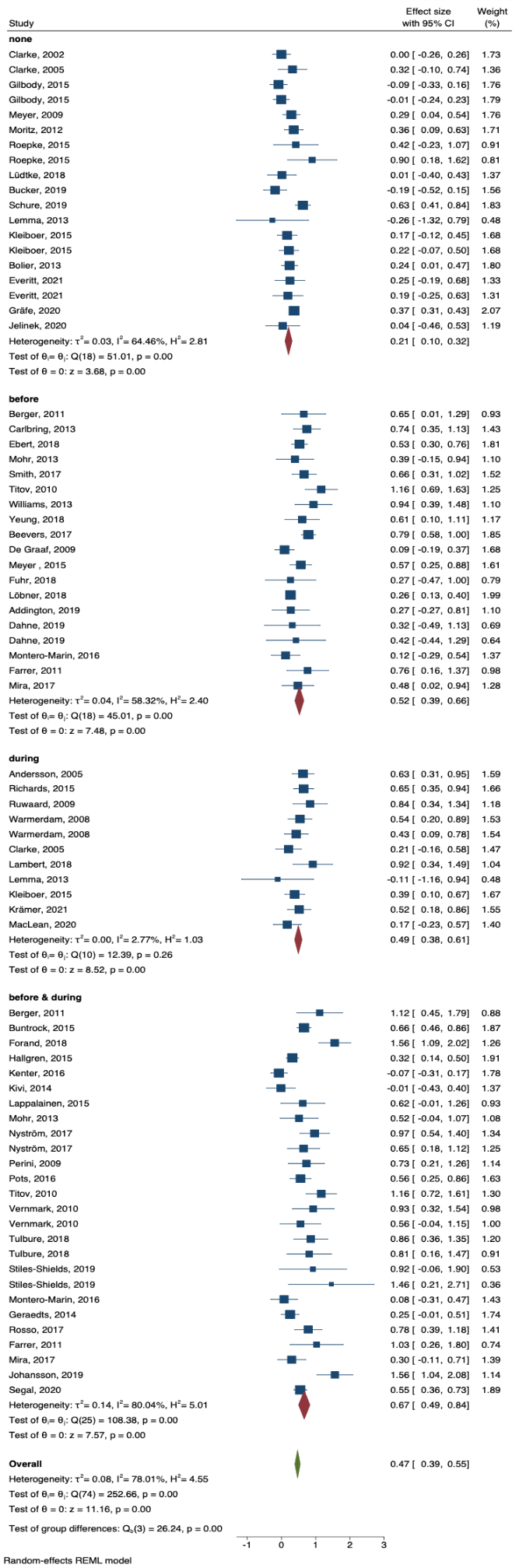

Supplement: Supplementary file 1 — Supplementary material [file mmc1.docx]
